# Review of the European *Greenomyia* Brunetti (Diptera, Mycetophilidae) with new descriptions of females

**DOI:** 10.3897/zookeys.77.936

**Published:** 2011-01-26

**Authors:** Olavi Kurina, Kjell Hedmark, Mats Karström, Jostein Kjærandsen

**Affiliations:** 1 Institute of Agricultural and Environmental Sciences, Estonian University of Life Sciences, Riia st 181, 51014 Tartu, Estonia; 2 Kungsfågelvägen 1C, S- 79432 Orsa, Sweden; 3 Älvvägen 4, S-96030 Vuollerim, Sweden; 4 Department of Biology, Museum of Zoology, Lund University, Helgonavägen 3, S-223 62 Lund, Sweden

**Keywords:** Mycetophilidae, *Greenomyia*, *Neoclastobasis*, fungus gnats, systematics, identification key, synonymy, Europe

## Abstract

The females of the four continental Greenomyia Brunetti species in Europe are associated with the males, diagnosed and keyed, providing the first association and description of the females of Greenomyia baikalica Zaitzev, 1994 and Greenomyia stackelbergi Zaitzev, 1982. Colour photographs of their habitus and line drawings of their female terminalia are provided. Greenomyia mongolica Laštovka & Matile, 1974 is found to be a senior synonym of Greenomyia theresae Matile, 2002. **syn. n.** The diagnostic characters used to distinguish between Greenomyia and Neoclastobasis Ostroverkhova in keys did not hold up to a closer scrutiny and leave the status of Neoclastobasis as separate genus questionable.

## Introduction

The genus Greenomyia Brunetti was erected to distinguish a single oriental species, Greenomyia nigricoxa Brunetti, 1912. Since then several species (mainly Holarctic) have been described and new combinations proposed. Laštovka and Matile (1974) and Chandler and Ribeiro (1995) characterised the genus and Matile (2002) provided a key to all 11 worldspecies of Greenomyia. Species of Greenomyia are mostly dark coloured medium-sized fungus gnats with a typical wing venation similar to that of Leia Meigen, 1818 and allied genera where R1 is notably shorter than the long and nearly horizontally aligned crossvein *rm*. Further diagnostic characters include the lateral ocelli being well separated from the eye margins and all veins reaching the wing margin (cf. Søli et al. 2000). Edwards (1925) introduced the tribe Leiini for Leia, Greenomyia and a number of other genera with an intermediate position between the subfamilies Sciophilinae and Mycetophilinae. Later the tribe has sometimes been given subfamily status (see review by Gammelmo 2004), but recent morphological and molecular studies have questioned its monophyly (e.g. Amorim and Rindal 2007; Rindal et al. 2009). Greenomyia appears most closely related to the genus Neoclastobasis Ostroverkhova, 1970, a genus that includes two eastern Palaearctic (Neoclastobasis sibirica Ostroverkhova, 1970 and Neoclastobasis kamijoi Sasakawa, 1964) and one European species (Neoclastobasis draskovitsae Matile, 1978). The genus Neoclastobasis is diagnosed by a prolonged apical palpal segment, the veins M_2_ and  CuA_1_ terminating before the wing margin, and distinctive terminalia (Ostroverkhova 1970; Matile 1978; Zaitzev 1994; Søli et al. 2000).

Species of Greenomyia are not frequently encountered in Europe and apart from the widespread Greenomyia mongolica Laštovka & Matile, 1974 (e.g. Bechev 1989; Ševčik and Martinovský 1999; Plassmann 1996; Caspers 1996; Kurina 1997; Chandler 2010), they are generally thought of as quite rare. A revitalized focus on fungus gnats the last two decades, however, has yielded a number of new records and five species are now reported from Europe: viz. Greenomyia baikalica Zaitzev, 1994, Greenomyia borealis (Winnertz, 1863), Greenomyia lucida (Becker, 1908), Greenomyia mongolica (= Greenomyia theresae Matile, 2002, syn. n.), and Greenomyia stackelbergi Zaitzev, 1982 (see Fig. 29 for published records, except Greenomyia lucida, which is endemic to the Canary Islands).

While the males of most Greenomyia species are adequately illustrated and keyed (Zaitzev 1982, 1994; Laštovka and Matile 1974; Chandler and Ribeiro 1995; Matile 2002) the females of several species remain to be properly diagnosed and described. The current communication was initiated by the finding of three Greenomyia species from two localities 1 km apart in Vuollerim, Lule Lappmark in northern Sweden (Kjærandsen et al. 2007). The material gave us the opportunity to associate and describe females of two species for the first time. The shifted focus to females further revealed that the generic characters separating Neoclastobasis from Greenomyia do not hold, and highlights a need to re-evaluate their status as separate genera.

## Material and methods

Material and collections from a wide range of Palaearctic sources were studied. The collecting methods, if known, are referred to in case by each specimen in the studied material section below. The following codens obtained from Evenhuis (2009) are used for depositories:

Coll. HedmarkPrivate collection of Kjell Hedmark, Orsa, Sweden.

Coll. SelinPrivate collection of Allan Selin, Tallinn, Estonia.

EIHUHokkaido University Museum, Sapporo, Japan.

IZBEInstitute of Agricultural and Environmental Sciences, Estonian University of Life Sciences (former Institute of Zoology and Botany), Tartu, Estonia.

MNHNMuseum National d’Histoire Naturelle, Paris, France.

MZLUMuseum of Zoology, Lund University, Lund, Sweden.

ZINZoological Institute of Russian Academy of Sciences, St. Petersburg, Russia.

ZMHBMuseum für Naturkunde Humbolt-Universität zu Berlin, Germany.

ZSMZoologische Staatssammlung in München, Germany

Three of four species were photographed and figured based on material collected in Vuollerim (Sweden), while illustrations of Greenomyia mongolica were based on Greek material. The terminalia were detached and cleared in a solution of KOH, followed by neutralization in acetic acid and washing in distilled water or in alcohol. The remaining chitinous parts were inserted into glycerine for detailed study, including illustration, and thereafter preserved as glycerine preparations in polyethylene micro vials. Habitus and wing photos were taken of specimens in alcohol, using a Canon 7D camera fitted with a Canon MP-E65 (F2.8 1–5 ×) lens. Illustrations of the terminalia were prepared using a U-DA drawing tube attached to a Olympus cx_3_1 compound microscope. Terminalia are figured in three different positions: laterally, dorsally and ventrally. Sternite VIII was detached and figured separately to better expose the shape of hypoproct and gonapophysis IX. The preservation method of the studied specimens is indicated in the material section for each species. We used a 70–80 % solution of ethanol for alcohol preservation and the chemical method described by Vockeroth (1966) for dry-mounting from alcohol. Slide mounting in Euparal followed the method described by Kurina (2008b). Morphological terminology follows Søli (1997).

## Systematics

### Key to females of European Greenomyia species

Compiled from (Zaitzev (1982, 1994), Chandler and Ribeiro (1995), Matile (2002) and original data.

**Table:** 

1	Wing hyaline (Fig. 12). Coxae yellow, femora and tibiae yellow, only hind femur brown in apical fourth. Mesonotum yellow with three fused longitudinal brown stripes. Cercus one segmented. Sternite VIII with three apical incisions (Fig. 28)	Greenomyia stackelbergi Zaitzev, 1982
–	Wing with apical or preapical dark band. Mesonotum dark brown to black. Coxae yellow or blackish. Cercus one- or two-segmented. Sternite VIII with single central incision apically (Figs 25–27)	2
2	Wing tip darkened on about apical third (Fig. 11)	3
–	Wing with preapical dark band leaving tip hyaline (Figs 9–10)	4
3	Mid and hind coxae brown to black (Fig. 3). Cercus clearly two-segmented	Greenomyia mongolica Laštovka & Matile, 1974
–	All coxae yellow or slightly darkened basally	Greenomyia lucida (Becker, 1908) [Endemic to the Canary Islands, not seen]
4	Last palpal segment elongated (Fig. 6). C terminating distinctly before apex of wing, making R_5_ straight to slightly sinuate (Fig. 10). Cercus two-segmented but segments partly fused (Figs 14, 18)	Greenomyia borealis (Winnertz, 1863)
–	Last palpal segment not elongated (Fig. 5). C terminating almost at apex of wing, making R_5_ distinctly arched (Fig. 9). Cercus one-segmented (Figs 13, 17)	Greenomyia baikalica Zaitzev, 1994

## The species

### Greenomyia baikalica

Zaitzev, 1994

**Figures 1–4.**
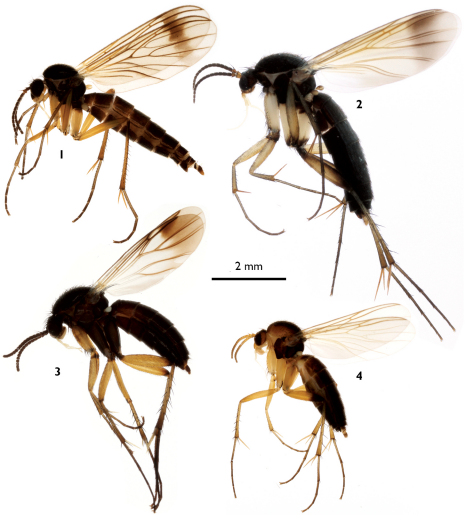
Habitus photos of European Greenomyia females. **1** Greenomyia baikalica **2** Greenomyia borealis **3** Greenomyia mongolica **4** Greenomyia stackelbergi.

Figures 1 5 9 13 17 21 25 

#### Material studied:

**SWEDEN.** 1♀, Lu. Jokkmokk, Vuollerim, Bomyrberget, in forest 135 m.a.s.l., Malaise trap, 16.–18.VIII.2006 (K. Hedmark leg.) [IZBE, mounted from alcohol]; 1♀, Lu. Jokkmokk, Vuollerim, Bomyrberget, 135 m.a.s.l., Malaise trap, 18.–24.VIII.2007 (K. Hedmark leg.) [MZLU, in alcohol]. **FINLAND.** 1♂, Kn. Sotkamo, Urpovaara, window trap N2, 26.VIII.–11.IX.1997 (M. Kuussaari leg.) [IZBE, on pin].

#### Diagnostic characters.

##### Female.

Thorax blackish, abdomen brown. Legs yellow, cx_3_ with small dark markings basally. All trochanters yellow, with small black apicoventral spots. Tibiae with dense brown setae. Scape and pedicel dark yellow, first flagellomere basally yellowish, rest of flagellum brown. Mouthparts pale yellow. Apical palpal segment 1.4–1.6 (n=2) times as long as penultimate segment. Wing with narrow preapical brownish band, gradually tapering towards hind margin. C terminating almost at apex of wing, R_5_ distinctly arched (Fig. 9). Medial and cubital veins both reach wing margin,  CuA_1_ basally obsolete, brownish shade along posterior margin of CuA2. Terminalia brown, cercus one-segmented, yellow apically. Tergite IX larger than tergite VIII. Gonapophysis IX visible in lateral view, with wide pear-shaped medial incision apically. Sternite VIII deeply incised apicomedially and moderately emarginated basally.

##### Male.

Coloration and other non-terminal characters including palpi similar to female. Apical palpal segment is 1.4 (n=1) times as long as penultimate segment.

**Figures 5–8.**
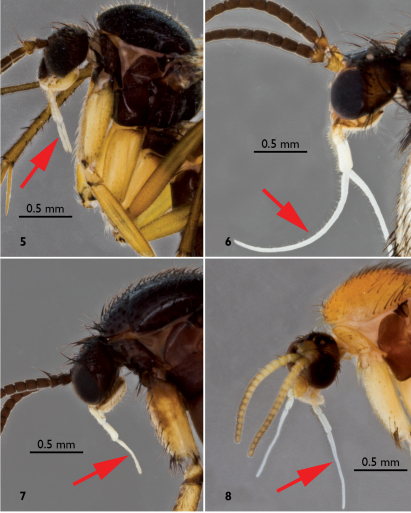
Head and palpi of European Greenomyia females, lateral view. **5** Greenomyia baikalica **6** Greenomyia borealis **7** Greenomyia mongolica **8** Greenomyia stackelbergi. Last palpal segment is indicated by a red arrow.

#### Remarks.

Zaitzev (1994) described this species from Siberian material (Buryat Republic). Subsequently only a few specimens have been recorded from Norway, Sweden, Finland and Russian Karelia (cf. Kjærandsen et al. 2007; Fig. 29). Zaitzev (1994) figured the male terminalia whereas the female terminalia have not been figured earlier. Using the key by Zaitzev (1994) the studied females run to Greenomyia baikalica and they are also morphologically conspecific with material of both sexes collected simultaneously in Russian Karelia (A. Polevoi, pers. comm.). The studied female specimens were collected in a boggy forest stand within a small (9 ha) protected remnant of semi-natural, mixed forest.

### Greenomyia borealis

(Winnertz, 1863)

Figures 2 6 10 14 18 22 26 

#### Material studied:

**SWEDEN**. 2♂♂, SK, Lund and Lund, Abusa, undated (J. W. Zetterstedt leg.) [MZLU, on pins]; 1♀, ÖG, Valdemarsvik, Snäckevarp (Snäckehvarps gästgifvaregård 1 km NE Gryt), 11 Aug 1825 (C. Stenhammar leg.) [MZLU, on pin]; 1♀, Lu. Jokkmokk, Vuollerim, in a garden, 105 m.a.s.l., Malaise trap, 8.–15.VIII.2008 (K. Hedmark and M. Karström leg.) [IZBE, mounted from alcohol]; **ESTONIA.** 1♂, Nigula NR, Haavapeaksi, sweeping, 12.VII.1998 (O. Kurina leg.) [IZBE, on pin]; 1♀, Tartu Marja 14, on window 21.VIII.2008 (O. Kurina leg.) [IZBE, on pin]. **GREECE.** 1♂ 2♀♀, Central Macedonia, Kerkini lakes area, village Vironia, Ramna site, 41°17'42.5"N, 023°11'33.1"E, 750 m.a.s.l., Malaise trap, 7.–13.VII.2008 (G. Ramel leg.) [IZBE, mounted from alcohol]; 1♂, Central Macedonia, Kerkini lakes area, village Vironia, Beabies site, 41°19'15.4"N, 023°13'39.6"E, 1150 m.a.s.l., Malaise trap, 21.–27.VII.2008 (G. Ramel leg.) [IZBE, mounted from alcohol]; 1♂ Central Macedonia, Kerkini lakes area, village Neo Petritsi, Midway site, 41°18'49.8"N, 023°16'35.6"E, 750 m.a.s.l., Malaise trap, 23.–29.VI.2008 (G. Ramel leg.) [IZBE, mounted from alcohol]. **KAZAKHSTAN.** 1♀, Alma-Ata, 13.–16.VI. 1824 (Kuzin leg.) [ZIN, on pin].

#### Diagnostic characters.

##### Female.

Thorax brown to blackish. Abdomen entirely brown or first two segments slightly lighter. Legs pale to yellow, except cx_2_ and cx_3_ with dark markings basally and apically, all trochanters brown and f_3_ brown, with lateral parts lighter to yellow. Tibiae with dense brown setae. Scape and pedicel dark yellow, flagellomeres brown. Mouthparts yellow. Apical palpal segment 5.0–5.4 (n=4) times as long as penultimate segment. Wing with broad preapical brownish band, reaching hind margin but gradually paler. C terminating distinctly before apex of wing, R_5_ straight to slightly sinuate (Fig 10). M_2_ and  CuA_1_ not reaching wing margin,  CuA_1_ basally obsolete or very weak. Terminalia brown; cercus yellow, two-segmented, apical segment small and partly fused with basal segment. Gonapophysis IX well sclerotized apically, visible in lateral view and with well developed narrow apical incision. Tergite VIII equal in size but slightly wider than tergite IX. Sternite VIII with medial incision apically and well emarginated basally.

##### Male.

Male. Coloration and other non-terminal characters including palpi similar to female. Apical palpal segment is 4.7–5.7 (n=4) times as long as penultimate segment.

**Figures 9–12.**
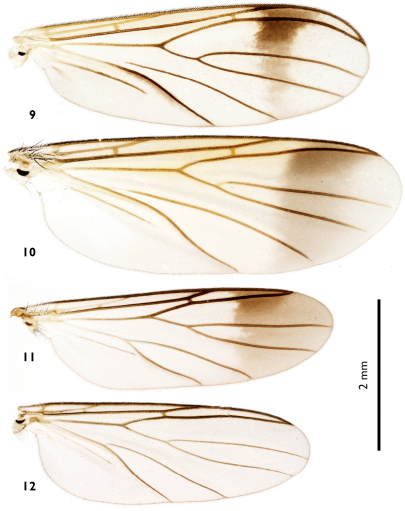
Wings of European Greenomyia females. **9** Greenomyia baikalica **10** Greenomyia borealis **11** Greenomyia mongolica **12** Greenomyia stackelbergi.

**Figures 13–16.**
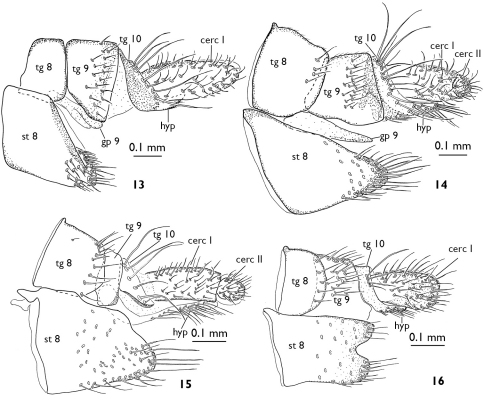
Female terminalia of European Greenomyia species, lateral view. **13** Greenomyia baikalica **14** Greenomyia borealis **15** Greenomyia mongolica **16** Greenomyia stackelbergi. cerc = cercus; gp = gonapophysis; hyp = hypoproct; st = sternite; tg = tergite.

**Figures 17–20.**
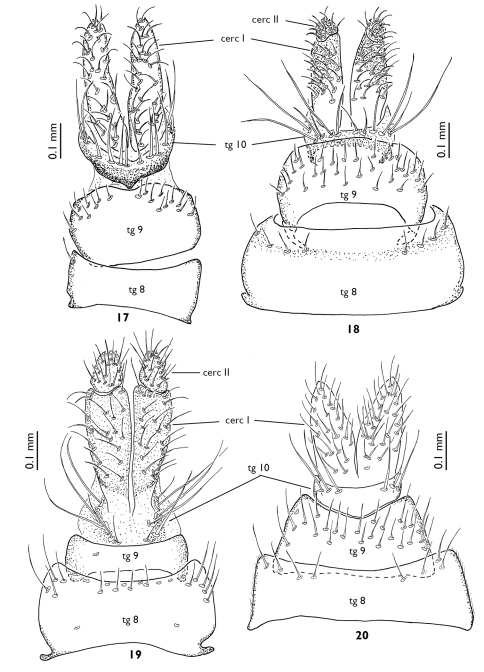
Female terminalia of European Greenomyia species, dorsal view. **17** Greenomyia baikalica **18** Greenomyia borealis **19** Greenomyia mongolica **20** Greenomyia stackelbergi.

**Figures 21–24.**
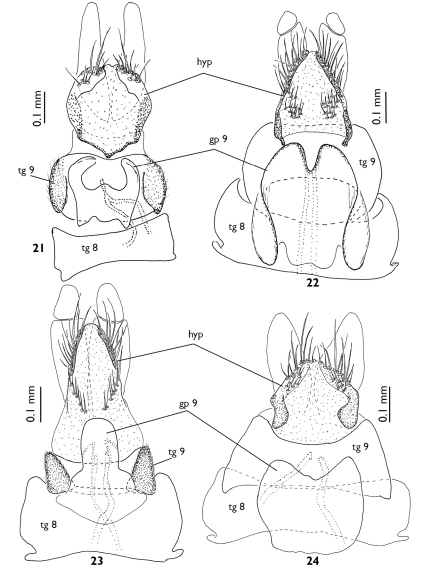
Female terminalia of European Greenomyia species, ventral view, sternite VIII detached. **21** Greenomyia baikalica **22** Greenomyia borealis **23** Greenomyia mongolica **24** Greenomyia stackelbergi.

**Figures 25–28.**
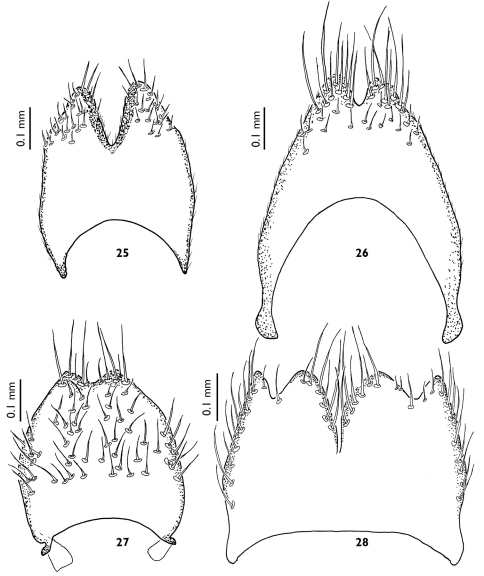
Female terminalia of European Greenomyia species, ventral view of sternite VIII. **25** Greenomyia baikalica **26** Greenomyia borealis **27** Greenomyia mongolica **28** Greenomyia stackelbergi.

#### Remarks.

While studying the Swedish specimen from Vuollerim, it ran by the first attempt using the key by Zaitzev (1994) to Neoclastobasis because of the extra long last palpal segment and M_2_ and  CuA_1_ not reaching the wing margin. The colouration of the studied specimen is, however, different and female terminalia lack strong spines on sternite VIII, being typical to all of the described Neoclastobasis species (Zaitzev 1982; JK and OK *pers. obs.* of Neoclastobasis kamijoi: 5♂♂ 2♀♀, South Korea, Sanan, I-li-Keumsan, [MNHN], 1♂ 3♀♀, Japan, Hokkaido, Sapporo [EIHU, MZLU]; Neoclastobasis draskovicae: paratypes, 1♂ 1♀ in MNHN, see Matile 1978). The discovery of a Greenomyia female, with similar size and coloration as the male of Greenomyia borealis in the same Malaise trap sample from northern Greece (Kerkini Lake area) allowed a safe association of the sexes. The females from Sweden, Estonia and Kazakhstan were further found to be conspecific with the Greek material of both sexes. According to Chandler et al. (2006), a male specimen from Greek mainland (Vikos Aoos National Park) has mainly yellow coxae, while other European specimens of Greenomyia borealis have mainly dark coxae. This may represent an intraspecific variation, however, all specimens studied during the current investigation have coxae whitish yellow. The above-mentioned Estonian specimen represents the first record of Greenomyia borealis from the country. The female specimen from Vuollerim was collected in the same garden as Greenomyia stackelbergi. Greenomyia borealis was previously known only with two 19th century findings from southern Sweden.

### Greenomyia mongolica

Laštovka et Matile, 1974

Figures 3 7 11 15 19 23 27

Greenomyia theresae  Matile, 2002, syn. n.
Greenomyia theresae Kurina 2008a: 255, 270.

#### Type material studied:

**Paratype** ♂ of Greenomyia mongolica: **MONGOLIA.** Central aimak, Tosgoni ovoo, 5–10 km N von Ulan-Baator, 1500–1700 m a.s.l., Exp. Dr. Z. Kaszab 1967 nr. 926, 19–24 Jul 1967 (Z. Kaszab leg.) [MNHN, JKJ-SPM-011843, on pin]

**Holotype** ♂ of Greenomyia theresae: **ITALY.** Aosta, Champlong, Dessus, 1000 m a.s.l., “courant sur Feuille de Frêne - 2m -”, 26 Aug 1997 (L. Matile leg.) [MNHN, JKJ-SPM-011844, on pin].

Other material studied: **SWEDEN.** 1♂, SÖ, Stockholm, Skarpnäck, Skarpa by, 13.VII.-4.X.2003 (B. Viklund leg.) [MZLU, in alcohol]. **GERMANY.** 1♂, D. Oberpfals, NM Main-Donau-Kanal (Proj. Warncke), 12.IX.–5.X.1988 (S. Blank leg.) [No. 30132 in ZSM, in alcohol]. **RUSSIA.** 2♂♂ 3♀♀, Nikolsk-Ussur, 29.VII.1926 (Kuznetzov leg.) [ZIN, on pins]. **ESTONIA.** 1♀, Kääriku, 5.X.1985 (H. Remm leg.) [IZBE, on pin]; 1♀, Luunja, 20.X.1996, on the house wall (O. Kurina leg.) [IZBE, on pin]; 6♂♂ 3♀♀, Karilatsi near Tartu, bait traps, 19.–28.VIII.2005 and 04.–25.IX.2005 (T. Tammaru leg.) [IZBE, on pins]. **HUNGARY.** 38♂♂ 3♀♀, 10 km S Eger, 47°49'11"N, 020°21'37"E, 20 Aug 1989 (R. Danielsson leg.) [MZLU, on pins]. **ITALY.** 1♂, Aosta valley, Verrayes, Promellian, 1200 m.a.s.l., sweeping, 17.VI.2007 (V. Soon leg.) [IZBE, on pin]; 1♀, Siena, 6.V.2007 (A. Selin leg.) [Coll. Selin, on pin]; 3♂♂, Trentino-Alto Adige, Prov. Bolzano, Parco Nationale dello Stelvia, Sulden Valley near Schmelz southwest of Prad, 46°36'42.1"N, 010°34'35.6"E, 940 m.a.s.l., 5.IX.–14.X.2005 (J. Ziegler and C. Lange leg.) [1♂ in ZMHB, 2♂♂ in IZBE, mounted from alcohol]. **GREECE.** 1♂ 2♀♀, Central Macedonia, Kerkini lakes area, village Vironia, Beabies site, 41°19'15.4"N, 023°13'39.6"E, 1150 m.a.s.l., Malaise trap, 30.VI.–6.VII.2008 (G. Ramel leg.) [IZBE, mounted from alcohol]; 6♀♀, Central Macedonia, Kerkini lakes area, village Vironia, Ramna site, 41°17'42.5"N, 023°11'33.1"E, 750 m.a.s.l., Malaise trap, 23.–29.VI.2008 (G. Ramel leg.) [IZBE, mounted from alcohol].

#### Diagnostic characters.

##### Female.

Female. Thorax dark brown to blackish. Abdomen entirely blackish brown or first three segments slightly paler laterally. cx1 entirely yellow or darkened in basal half, cx_2_ and cx_3_ entirely dark brown to black. Fore trochanter yellow basally, brown apically. Mid and hind trochanters brown. f_1_ and f_2_ yellow, f_3_ yellow with brown apical fifth. Tibiae yellow, apically slightly darkened, with dense brown setae. Scape, pedicel and flagellomeres brown. Mouthparts pale yellow. Apical palpal segment 1.8–2.2 (n=5) times as long as penultimate segment. Wing tip shaded on about apical third, with darkened area along fore margin. All veins reach wing margin, M_2_ sometimes basally obsolete or very weak, A_1_ ending close to, sometimes fused into base of CuA2. Terminalia brown. Cercus distinctly two-segmented, apical segment small, ovate. Gonapophysis IX membranous, widely protruding apically, not visible in lateral view. Tergite VIII larger than tergite IX. Sternite VIII apically with shallow medial incision, moderately emarginated basally.

##### Male.

Male. Coloration and other non-terminal characters including palpi similar to female. Apical palpal segment is 1.7–2.1 (n=5) times as long as penultimate segment.

#### Remarks.

This species was originally described by Laštovka and Matile (1974) based on Mongolian material and subsequently widely recorded in Europe. Chandler (2005) did not include Greenomyia mongolica in the European list and assigned all records to Greenomyia theresae, a species described from northern Italy by Matile (2002). Careful comparisons of type material of both species at MNHN in Paris (independently undertaken by two of the authors, OK and JK; the holotype of Greenomyia mongolica deposited in the Hungarian Natural History Museum was not available for the study) did not indicate any substantial differences in their male terminalia. The minor diagnostic characters as indicated in the original description and illustrations by Matile (2002) are liable to different angles of views only. Consequently we have come to the conclusion that Greenomyia theresae at present state of knowledge must be treated as a junior synonym of Greenomyia mongolica and that all published records in Europe should rather be associated with the latter. In addition to the studied type material, we also compared the terminalia of female specimens from the Russian Far East, Estonia and northern Greece without finding any reliable differences. Moreover, Papp (2000) confirmed conspecificity when he compared central European material from Hungary with the Mongolian type material. Male terminalia are figured by Laštovka and Matile (1974) and subsequently by Matile (2002), while female terminalia have previously been figured by Zaitzev (1982) and Kurina (1997). Our association of males and females are based on multiple simultaneous findings in trap samples (see above) that agrees with previous descriptions of the female. In the Pre-Balkan mountain range in Bulgaria, the species has been collected in xerotermic oak forest (Bechev 2000). The species was quite common in samples taken in a bait trap, operated on the basis of a mixture of fermenting sugar and red wine, in southern Estonia (see also Kurina 2006). The above-mentioned specimens from Greece are the first records from the country.

### Greenomyia stackelbergi

Zaitzev, 1982

Figures 4 8 12 16 20 24 28 

#### Type material studied:

**Holotype** ♂, **RUSSIA.** Primorskiy Terr., Santaheza, 07.VII.1927 (A. Stackelberg leg.) [ZIN, on pin].

Other material studied: **SWEDEN.** 4 ♀♀, Lu. Jokkmokk, Vuollerim, in garden, 105 m.a.s.l., Malaise trap, 11.VIII.–19.IX.2003 (K. Hedmark and M. Karström leg.); 1♂, the same locality, Malaise trap 11.–19.VIII.2004; 25♂♂ 15♀♀, the same locality, Malaise trap 11.VIII.–7.X.2005; 11♂♂ 4♀♀, the same locality, Malaise trap 11.VIII.–22.IX.2006; 18♂♂ 14♀♀, the same locality, yellow pan-trap VII–08.X.2006; 17♂♂ 7♀♀, the same locality, yellow pan-trap 16.VI.–20.VII.2007; 14♂♂ 4♀♀, the same locality, Malaise trap 12.VIII.–28.IX.2007; 9♂♂ 3♀♀, the same locality, window trap VI–11.IX.2007; 4♂♂ 1♀, the same locality, Malaise trap 13.–27.VI.2008; 1♀, the same locality, yellow pan-trap 19.VI.2008; 1♂, the same locality, window trap 1.VI.–1.VII.2008. In total 153 specimens: 100♂♂ 53♀♀, [most in Coll. Hedmark, some in IZBE and MZLU, most of the material preserved in alcohol, while some specimens are mounted from alcohol to pins or slide mounted].

#### Diagnostic characters.

##### Female.

Female.Thorax bi-coloured; mesonotum yellow with variably developed black thoracic stripes; pronotum and propleuron yellow, other pleural parts brown to blackish. Abdominal sternites I-IV entirely yellow or slightly brownish; tergites of first four segments bi-coloured: basally yellow, apically brown (in a few occasions first four tergites entirely brown). Legs all yellow except dark brown band on apical fourth of hind femur. Tibiae densely covered with brown setulae. Scape, pedicel, and 3–5 flagellomeres yellow, rest of flagellum light brown. Mouthparts yellow. Apical palpal segment 4.1–4.4 (n=5) times as long as penultimate segment. Wing hyaline with slight yellowish tinge, all veins reach wing margin, M1 and CuA2 basally obsolete or very weak. Terminalia brown, cercus one-segmented, apically yellow. Gonapophysis IX membranous, subsquare with shallow incision apically, not visible in lateral view. Tergite VIII wider than tergite IX. Sternite VIII medially with deep and narrow incision, lateral incisions more shallow.

##### Male.

Male. Coloration and other non-terminal characters similar to female. The apical palpal segment is 4–5 (n=5) times as long as penultimate segment.

#### Remarks.

**Figure 29.**
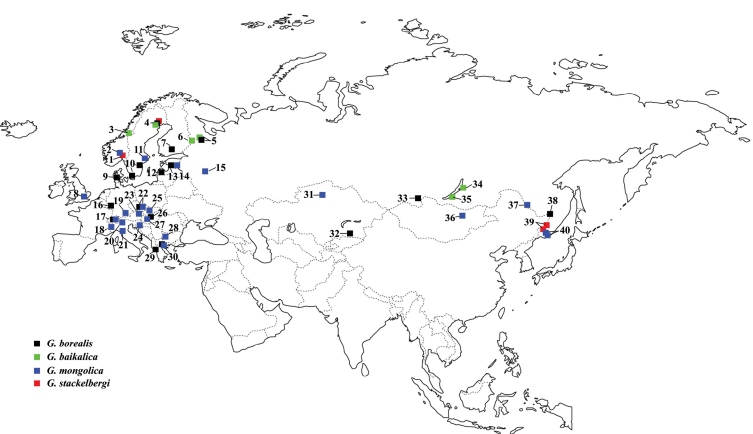
Known records of European Greenomyia species. Greenomyia borealis (black squares): **4** original data **5** Polevoi 2000 (marked with a question mark) **7** Chandler 2005 **9** Chandler 2005 **10** Kjærandsen et al. 2007, original data **12** Lackschewitz 1937 **13** original data from two localities **16** Winnertz 1863 **17** Chandler 1998, 2005 (doubtful) **22** Ševčík and Košel 2009 **26** Ševčík and Papp 2001 **29** Chandler et al. 2006 **30** original data **32** Zaitzev 1982, 1994 **33** Zaitzev 1982, 1994 **38** Zaitzev 1982, 1994. Greenomyia baikalica (green squares): **3** Gammelmo and Søli 2006 **4** Kjærandsen et al. 2007 **5** Polevoi 2000 (three different localities) **6** Polevoi 2001 **34** Zaitzev 1994 **35** Zaitzev 1994. Greenomyia mongolica (blue squares): **2** Søli et al. 2009 **8** Chandler 2010 (seven different localities) **11** Kjærandsen et al. 2007 **14** Kurina 1997 **15** Zaitzev 1994 **17** Matile 2002 (as Greenomyia theresae) **18** Matile 2002 (as Greenomyia theresae) **19** Caspers 1996 **20** Kurina 2008a (as Greenomyia theresae) **21** original data **22** Martinovský and Ševčik 1998, Martinovský and Barták 2000 **23** Plassmann 1996 **24** Matile 2002 (as Greenomyia theresae) **25** Ševčík and Martinovský 1999 **27** Papp 2000 (several different localities), original data **28** Bechev 1989 **30** original data **36** Laštovka and Matile 1974 **37** Zaitzev 1982, 1994 **40** Zaitzev 1982, 1994. Greenomyia stackelbergi (red squares): **1** Søli and Kjærandsen 2008 **4** Kjærandsen et al. 2007 **39** Zaitzev 1982, 1994.

Besides its peculiar distribution (see Fig. 29), Greenomyia stackelbergi is unique among the four studied species in having vivid yellowish colouration and hyaline wings. It was described from South Primorje in the Russian Far East (Zaitzev 1982) and has subsequently been recorded only from two semi-urban localities in the Nordic region: the single locality in Swedish Lapland (present material, Kjærandsen et al. 2007) and from one locality in the capital of Norway, Oslo (Søli and Kjærandsen 2008). Eight years of collecting (2002–2009) with Malaise traps, yellow pan-traps and window traps near a compost in the garden of one of the authors (MK) yielded 153 specimens, indicating rise and decline of a small population. None was collected in the first and the last year, while four in 2003, one in 2004, 40 in 2005, 47 in 2006, 54 in 2007 and seven in 2008. The flight activity lasted almost the whole vegetation season, from the middle of June (in 2007) to the beginning of October (in 2004). A garden compost is the supposed microhabitat for this population of Greenomyia stackelbergi and its origin should be somewhere in the surroundings. A close potential natural habitat could be the Vuollerim ravine a few hundred meters away. Waste from picked forest fungi might be another possibility**.**

## Discussion

Species descriptions of fungus gnats are largely based and depending on characters in the male terminalia. Females are often ignored in taxonomic reviews and only a few generic reviews cover all or the majority of associated females (e.g. Søli 1997; Martinsen and Søli 2000; Kjærandsen 2006, 2009). Still, females usually have distinctive yet less pronounced characters in their terminalia. In the case of the few European Greenomyia species we found it fairly easy to safely associate the females based on body characters such as colouration patterns, wing shape and venation details shared between the sexes, and the associations were further strengthened by co-occurrence in multiple trap samples.

Our study of Greenomyia revealed that the diagnostic characters used to distinguish Greenomyia and Neoclastobasis in keys (e.g. Søli et al. 2000) does not hold up to a closer scrutiny, especially when both sexes are considered. Both sexes of Greenomyia borealis havewings where M_2_ and  CuA_1_ end slightly before the wing margin, and both sexes of Greenomyia borealis and Greenomyia stackelbergi have prolonged apical segment of their palps. These characters are akin to those used to diagnose Neoclastobasis. Yet, although the three known species of Neoclastobasis are very similar to Greenomyia in general appearance they show distinctive features in their terminalia that separate them from Greenomyia. In Greenomyia the dorsal branch of the male gonostylus always has two distinct combs of blunt spines on an otherwise bare inner surface. In Neoclastobasis the entire inner surface is covered with short blunt setae and a single row of larger spines is situated basally. Females of Neoclastobasis have a few short and strong spines along the apical margin of sternite VIII, which are never found in Greenomyia. We think Greenomyia and Neoclastobasis may prove to be monophyletic sistertaxa, but pending genetic studies and a better definition of the entire Leiini clade we leave the question whether they deserve to retain their status as separate genera or could be joined into one. In the meantime separating the two genera must rest entirely on differences in their terminalia as described above.

## Supplementary Material

XML Treatment for Greenomyia baikalica

XML Treatment for Greenomyia borealis

XML Treatment for Greenomyia mongolica

XML Treatment for Greenomyia stackelbergi
